# On the origin of the shift between vertical excitation and band maximum in molecular photoabsorption

**DOI:** 10.1007/s00894-020-04355-y

**Published:** 2020-04-21

**Authors:** Shuming Bai, Ritam Mansour, Ljiljana Stojanović, Josene M. Toldo, Mario Barbatti

**Affiliations:** 1 Aix Marseille University, CNRS, ICR, Marseille, France; 2 Department of Chemistry, Duke University, Durham, NC 27708 USA; 3 School of Biological and Chemical Sciences, Queen Mary University of London, Mile End Road, London, E1 4NS UK

**Keywords:** Spectrum simulation, Vertical excitations, Excited states, Absorption cross-section

## Abstract

The analysis of the photoabsorption spectra of molecules shows that the band maximum is usually redshifted in comparison to the vertical excitation. We conducted a throughout analysis of this shift based on low-dimensional analytical and numerical model systems, showing that its origin is rooted in the frequency change between the ground and the excited states in multidimensional systems. Moreover, we deliver a benchmark of ab initio results for the shift based on a comparison of vertical excitations and band maxima calculated with the nuclear ensemble approach for the 28 organic molecules in the Mülheim molecular dataset. The mean value of the shift calculated over 60 transitions is 0.11 ± 0.08 eV. The mean value of the band width is 0.32 ± 0.14 eV.

**Electronic supplementary material:**

The online version of this article (10.1007/s00894-020-04355-y) contains supplementary material, which is available to authorized users.

**Graphical abstract:**
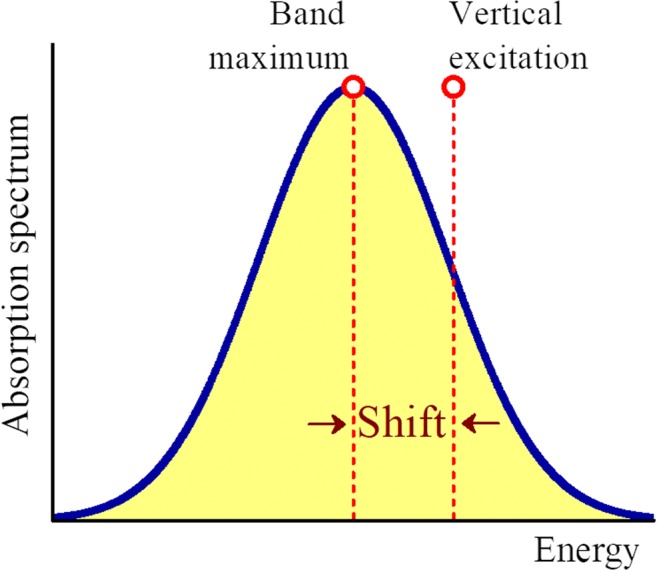
.

## Introduction

**Fig. 1:**
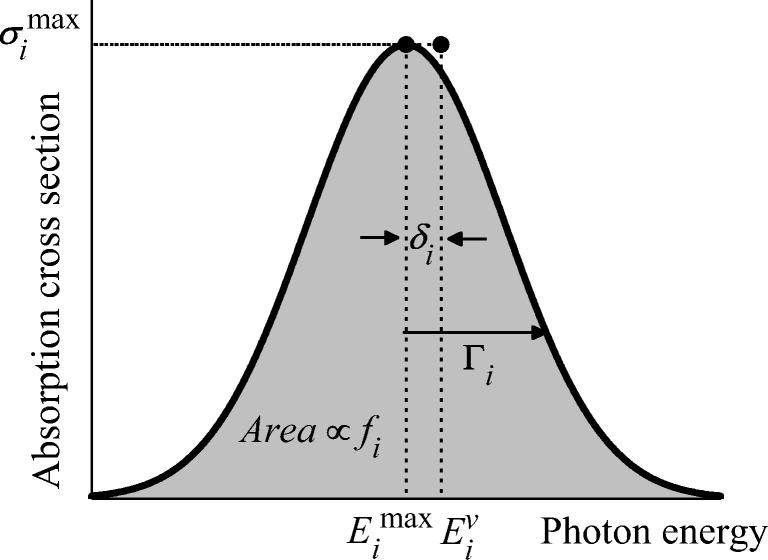
Schematic illustration of the absorption band parameters

In the computational analysis of UV/Vis photoabsorption spectra of molecules, several authors have noticed that the band maximum ${E}_i^{\mathrm{max}}$ and the vertical excitation ${E}_i^v$ of band *i*, computed at the same electronic structure level, are shifted by up to 0.2 eV, with the band maximum systematically redshifted [1–5]. This shift *δ*_*i*_ (Fig. 1) is a parameter that plays a particularly important role in the comparison between theory and experiments and in the evaluation of the precision of computational methods [4]. Therefore, a direct experimental/computational comparison without considering the shifts into account may lead to systematic errors.

In the last years, diverse methods for first-principle spectrum simulations have been proposed [5–10], leading to high accurate predictions of band shapes, including their vibrational resolution [11]. Through simulations of vibrationally resolved absorption spectra of several molecules, Avila Ferrer et al. [3] confirmed the existence of the redshift and suggested to compare vertical excitations not to the band maximum but to the first moment *M*^1^ (center of gravity) of the absorption band. Nevertheless, to the best of our knowledge, the origin of the redshift has not been clarified so far. We know from our experience with spectrum simulations based on the nuclear ensemble approach [12] that the redshift also occurs when the band maximum is simulated with incoherent transition processes. Therefore, its origin should have an underlying semiclassical mechanism, not directly related to quantum effects.

**Fig. 2:**
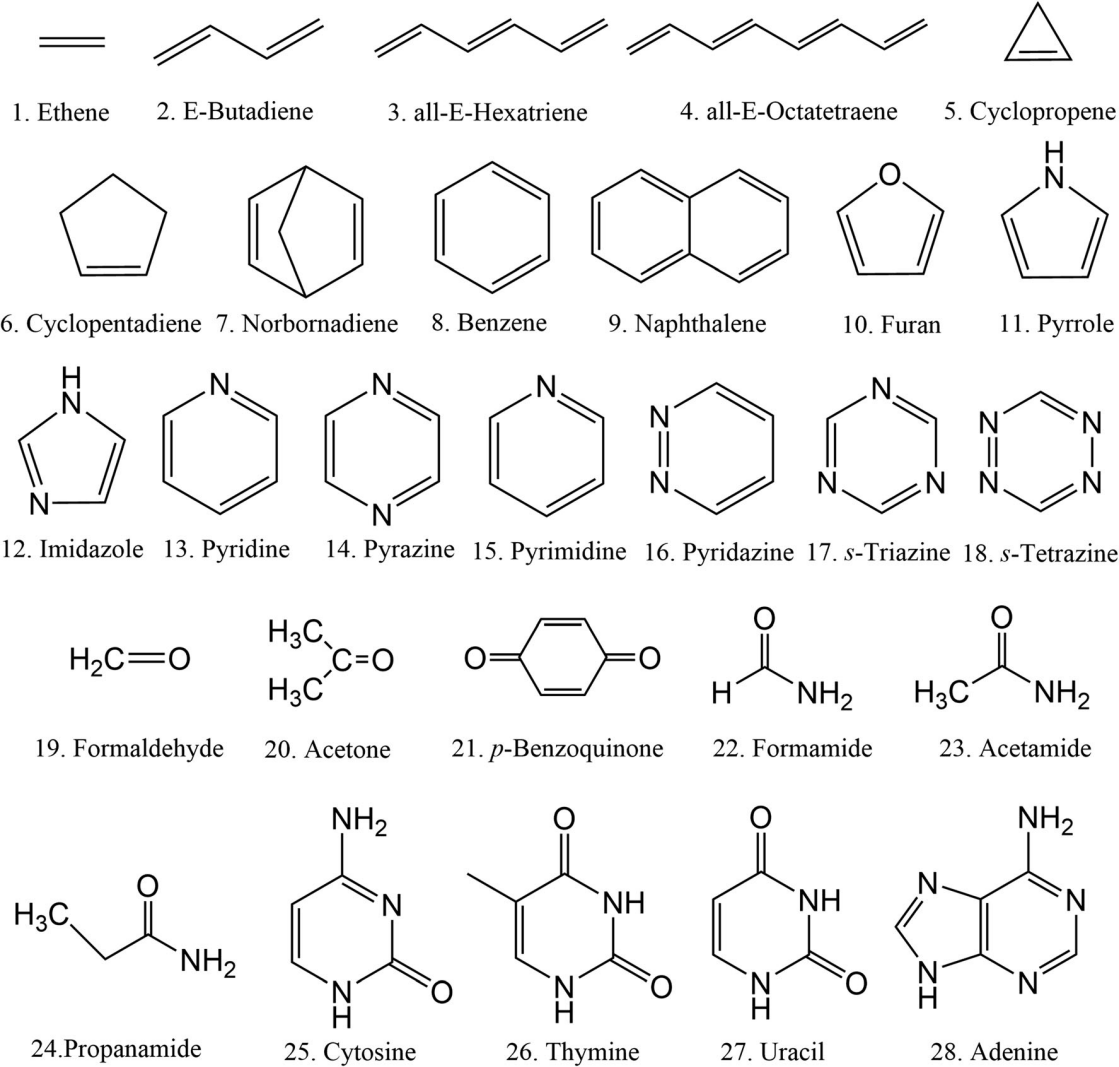
Structures of the 28 molecules in the Mülheim dataset

Using a simple analytical model, we show here that when a multidimensional ground-state vibrational wave function is projected onto the excited state, the frequency change between the excited and the ground states causes the maximum spectral intensity to be displaced to lower energies compared to the vertical excitation. We confirmed such dependence of the shift on the frequency change using low dimensionality numerical models. Finally, we deliver a benchmark of shift values for the first bands of all 28 organic molecules in the Mülheim molecular dataset [13] (Fig. 2). This benchmark was produced comparing ab initio vertical excitations to band maxima simulated with the nuclear ensemble approach at the same electronic structure level.

## Computational details

The ground state optimization and normal mode analysis of all 28 molecules were done with the resolution of the identity coupled-cluster to approximated second order (RI-CC2) [14, 15] with the cc-pVTZ basis set. The Cartesian coordinates of the ground state minimum geometries are given in the supporting information. Excited states were calculated at RI-CC2 level with the aug-cc-pVDZ basis set [16]. In a few cases with convergence problems, we alternatively employed the algebraic diagrammatic construction to second order (ADC(2)) [17].

For the nuclear ensemble simulations [12], the nuclear configurations were sampled according to a harmonic oscillator Wigner distribution based on normal modes in the ground state minimum. An ensemble of 500 nuclear geometries was generated for each molecule of the dataset. For each nuclear configuration, up to 15 electronic states were computed using RI-CC2/aug-cc-pVDZ. Some of the molecules presented recurrent convergence problems with CC2, caused by near-degenerated excited states. In such cases, we employed RI-ADC(2)/aug-cc-pVDZ in the spectrum simulation. Each spectrum was plotted with a Gaussian linewidth broadening of 0.05 eV. Details on vertical excitations and simulated spectra are given in the supporting information.

RI-CC2 and RI-ADC(2) calculations were done with TURBOMOLE [18]. Nuclear ensemble spectra were done with NEWTON-X [19, 20] interfaced with TURBOMOLE. A program to calculate spectra based on Eq. (13) is freely available for download [21].

## Results and discussion

### Hypotheses to explain the band maximum shift

To understand the shift *δ*_*i*_ between the vertical excitation and the band maximum, it is useful to analyze it in terms of the nuclear ensemble approach [12]. In this method, the absorption spectrum is built as an incoherent sum of transitions from nuclear geometries representing the vibrational distribution of the electronic ground state. Thus, the nuclear ensemble approach delivers a band envelope without vibrational resolution but with enough information to estimate the shift.

To determine the origin of the shift, we considered three possible causes: (1) the oscillator strength variation in the sampling (a post-Condon effect), (2) the displacement of the vibrational mode in the excited state, and (3) the vibrational frequency change between the ground and excited states. The penetration of the wave function in regions beyond the classical return point may also cause a shift, but it tends to be negligible for large molecules [22].

The post-Condon effect (1) was discarded after we noticed that nuclear ensemble simulations using an uniform value for the oscillator strength still yields the shift. We show in the next sections that the normal mode displacement (2) also does not cause the shift, although it impacts its value. Finally, we found out that the frequency change between the ground and excited states (3) is at the root of the phenomenon. The underlying reason for the shift between the vertical excitation and the band maximum is that for a multidimensional potential energy surface, although most of the nuclear geometries are distributed around the equilibrium geometry, there are even more geometries displaced from equilibrium satisfying the resonance condition Δ*E*_*ge*_ = *E*_*P*_ (Δ*E*_*ge*_ is the energy gap between the ground and the excited states, and *E*_*P*_ is the photon energy).

### Analytical analysis of the shift origin

To understand the origin of the shift, consider the following simple two-mode/two-state analytical model. The two states are separated by a vertical transition energy *E*^*v*^. For each state, the potential energy surface is supposed to be given by two uncoupled harmonic and degenerated potentials in the coordinates *q*_1_ and *q*_2_, with the ground and excited states sharing the same equilibrium geometry, but with different angular frequencies *ω*_*g*_ and *ω*_*e*_. Without losing generality, the energy of the ground-state equilibrium geometry is assumed to be zero. Thus, the ground- and excited-state potential energies are given as :

1 $$ {\displaystyle \begin{array}{l}{E}_g=\frac{1}{2}{\mu \omega}_g^2\left({q}_1^2+{q}_2^2\right),\\ {}{E}_e={E}^v+\frac{1}{2}{\mu \omega}_e^2\left({q}_1^2+{q}_2^2\right).\end{array}} $$

where *μ* is the reduced mass. The resonance condition is

2 $$ \Delta {E}_{ge}-{E}_P=\left({E}^v-{E}_P\right)+\frac{1}{2}\mu \left({\omega}_e^2-{\omega}_g^2\right)\left({q}_1^2+{q}_2^2\right)=0. $$

In a semiclassical approximation, the spectrum is given by [23]

3 $$ \sigma =k\int \rho \left({q}_1,{q}_2\right)g\left(\Delta {E}_{eg}\left({q}_1,{q}_2\right)-{E}_p\right)\kern0.1em {dq}_1{dq}_2 $$

where *ρ* is the distribution of geometries in the ground state, and *g* is a line-shape function satisfying the resonance condition in Eq. (2). *k* is a constant, which includes the oscillator strength between the two states (Condon approximation). If *ρ* is given by a Wigner distribution for the quantum harmonic oscillator,

4 $$ {\displaystyle \begin{array}{l}\rho \left({q}_1,{q}_2\right)=\left(\frac{\alpha }{\pi}\right)\exp \left(-\alpha \left({q}_1^2+{q}_2^2\right)\right),\\ {}\alpha \equiv \frac{{\mu \omega}_g}{\mathrm{\hslash}},\end{array}} $$

and the line-shape function is taken as a normalized Gaussian function with arbitrary width *w*,

5 $$ g(x)=\frac{1}{w\sqrt{\pi }}\exp \left(-\frac{x^2}{w^2}\right), $$

we obtain

6 $$ \sigma =\frac{k\alpha}{w{\pi}^{3/2}}\int \exp \left(-\alpha \left({q}_1^2+{q}_2^2\right)-\frac{1}{w^2}{\left(\left({E}^v-{E}_p\right)+\frac{1}{2}\mu \left({\omega}_e^2-{\omega}_g^2\right)\left({q}_1^2+{q}_2^2\right)\right)}^2\right)\kern0.1em {dq}_1{dq}_2. $$

*w* is assumed to be much narrower than the band width, and in the limit when it tends to zero, *g* converges to a Dirac delta function.

In polar coordinates (*q*_1_ = *R* cos(*θ*), *q*_2_ = *R* sin(*θ*)), Eq. (6) becomes

7 $$ \sigma =\frac{k\alpha}{w{\pi}^{3/2}}\int \exp \left(-\alpha {R}^2-\frac{1}{w^2}{\left(\left({E}^v-{E}_p\right)+\frac{1}{2}\mu \left({\omega}_e^2-{\omega}_g^2\right){R}^2\right)}^2\right)\kern0.1em RdRd\theta, $$

which can be analytically integrated to give

8 $$ \sigma =-\frac{k}{A}\exp \left(\frac{w^2}{A^2}\left(1+\frac{2A}{w^2}\left({E}^v-{E}_p\right)\right)\right)\left(1+\mathit{\operatorname{erf}}\left(\frac{w}{A}\left(1+\frac{A}{w^2}\left({E}^v-{E}_p\right)\right)\right)\right), $$

where

9 $$ A=\frac{\mathrm{\hslash}\left({\omega}_e^2-{\omega}_g^2\right)}{\omega_g}. $$

The maximum of this spectral band happens for the photon energy *E*^max^ satisfying *dσ*/*dE*_*P*_ = 0. If we expand this derivative to the first order in *E*_*p*_ around *E*^*v*^, we obtain the shift

10 $$ \delta \equiv {E}^v-{E}^{\mathrm{max}}=\frac{\mathrm{\hslash}}{2}\frac{\left({\omega}_g^2-{\omega}_e^2\right)}{\omega_g}. $$

Because upon excitation the bond is loosened, it implies that *ω*_*e*_ < *ω*_*g*_and, therefore, *E*^*v*^ > *E*^max^. This means that the band maximum is redshifted in relation to the vertical excitation.

**Fig. 3:**
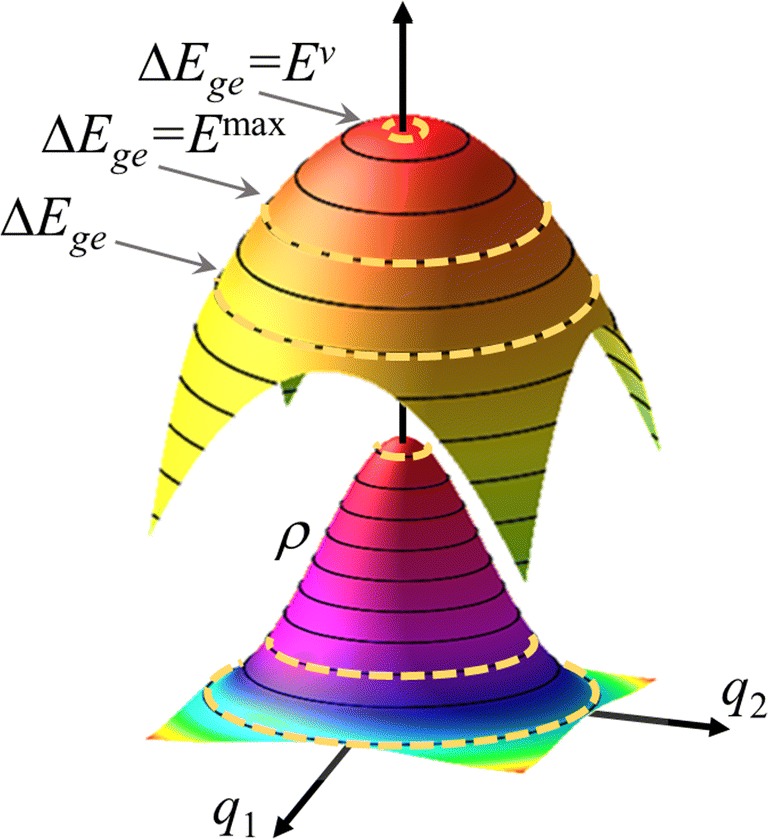
Illustration of the 2-mode/2-state analytical model. The upper surface shows the potential energy difference Δ*E*_*ge*_, while the bottom surface illustrates the Wigner distribution *ρ* in the ground state. The dashed curves indicate isoenergetic curves satisfying the resonance condition Δ*E*_*ge*_ = *E*_*P*_

With this simple model, it is easy to understand the origin of the shift. For a certain photon energy *E*_*P*_, the spectral intensity in Eq. (3) is determined by both the ground state population on the isoenergetic line satisfying the resonance condition and the length of this line (Fig. 3). In this model, this line is merely a circle, but it is a hyperline in general multidimensional cases. In one limiting case, the ground state population is maximal (*q*_1_ = *q*_2_ = 0) but the line length is too short, yielding a low spectral intensity. In the case of excited and ground states sharing the same equilibrium geometry, this limit happens at *E*^*v*^. In the other limiting case, the curve length tends to infinite (*q*_1_ = *q*_2_ → ∞), but the ground state population is null, also yielding a minimal intensity. Between the two extremes, the band maximum occurs at *E*^max^, with optimal length and population values, which is proportional to the difference between the squared frequencies of the excited and ground states (Eq. (10)).

Although there are no analytical results for multidimensional, realistic cases, this dependence of the shift on the difference of the squared frequencies seems to be general. For instance, as mentioned in the introduction, Avila Ferrer et al. [3] proposed to compare vertical excitations to the first moment *M*^1^ of the absorption band to approximately account for the shift effect. They recall that the *M*^1^ can be analytically computed for harmonic ground and excited states in the Franck-Condon approximation, with the excited state built in the vertical Hessian approach. In this model, the shift between the vertical excitation and *M*^1^ is [24]

11 $$ {E}^v-{M}^1=\sum \limits_r\frac{\mathrm{\hslash}}{4}\frac{\left({\omega}_{g,r}^2-{\omega}_{e,r}^2\right)}{\omega_{g,r}}\coth \left(\frac{\mathrm{\hslash}{\omega}_{g,r}^2}{2{k}_BT}\right), $$

where *k*_*B*_ is the Boltzmann constant, *T* is the temperature, and *r* runs over the normal modes. In the limit of low temperature and two degenerated normal modes, this expression yields the shift given by Eq. (10).

Note that the shift seems to be a multidimensional phenomenon. We have not been able to find an analytical expression for the shift in a single-dimensional model, but numerical estimates showed that in such case, the shift is a function of the width *w*, and it tends to zero for small *w*. In the limit of a Dirac delta function (*w* = 0), the band of the single-dimensional model is peaked at the vertical excitation [23].

### Analysis of the shift with model systems

To get further numerical insights into the origin of the shift, we simulated the absorption spectrum of a model system with few vibrational modes using the nuclear ensemble approach. The advantage of working with such low dimensionality systems is that we can systematically control the values of key parameters to verify their impact on the spectrum.

The model systems are composed of two harmonic potentials representing the ground and the first excited states. The simplest model contains a single vibrational mode (1 V) with ground state frequency *ω*_*g*_ and excited state frequency given by ${\omega}_e^2/{\omega}_g^2=\eta$, where *η* is a constant. The potential energies of the ground and excited states are

12 $$ {\displaystyle \begin{array}{l}{E}_g=\frac{1}{2}{\mu \omega}_g^2{q}^2\\ {}{E}_e={E}^v+\frac{1}{2}{\mu \eta \omega}_g^2{\left(q-d\right)}^2\end{array}} $$

In the simulations, *E*^*v*^ = 4.08 eV (0.15 au), *μ* = 1.0 amu (1823 au), and *ω*_*g*_ = 1500 cm^−1^ (0.0068 au). To compute the spectrum, 50,000 geometries were sampled from a Wigner distribution for *E*_*g*_, and the oscillator strength was assumed to be 1.0 for all points.

As expected for the one-dimensional model, the results do not show any shift for small *w*.

We extended the 1 V model into three vibrational modes (3 V), with ground-state frequencies *ω*_*g*1_ = 1800, *ω*_*g*2_ = 1500, and *ω*_*g*3_ = 1000 cm^−1^; frequency-change parameters *η*_1_, *η*_2_, and *η*_3_; and displacements *d*_1_, *d*_2_, and *d*_3_. All other parameters were kept at the same values as in the 1 V model.

The results for the shift with the 3 V model are given in Table 1. Now, we see that displacements without a frequency change lead to a negligible shift. For *η*_2_ = 0.8, as the displacements of the other two modes increase, the shift increases, too. This result tells that although the displacement does not cause the shift, it modulates the shift value.

**Table 1:** Numerical simulations of the shift (eV) between the vertical excitation and the band maximum for a model with three vibrational modes, considering a shift between the ground and excited-state frequencies (given by *η*_*ι*_) and a displacement *d*_*i*_ (au) between the modes. All results with *η*_1_ = *η*_3_ = 1.0 and *d*_2_ = 10 au

*η*_2_	*d*_1_	*d*_3_	Shift/eV
1.0	3.0	3.0	− 0.01
0.8	0.0	0.0	0.08
0.8	3.0	3.0	0.15
0.8	8.0	8.0	0.27
0.8	10.0	10.0	0.31
0.8	15.0	15.0	0.39

### Benchmark of shift values

**Fig. 4:**
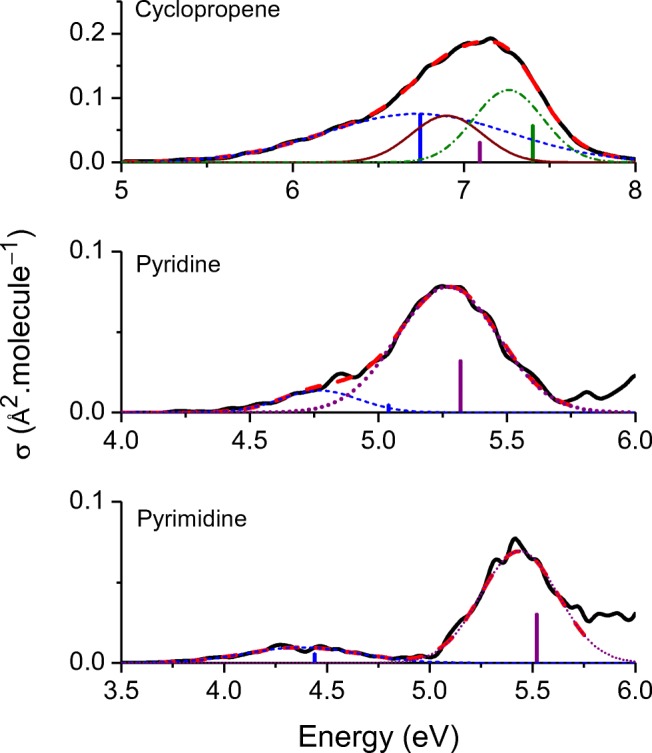
Comparison between the nuclear ensemble spectrum simulation (solid black) and the vertical excitations (sticks) for (top) cyclopropene, (middle) pyridine, and (bottom) pyrimidine. The fitting of the nuclear ensemble result is shown as a dashed red curve. The Gaussian function components of the fitting, corresponding to the sub-bands of each vertical excitation, are shown as well

Based on the vertical excitations and the results from the nuclear ensemble simulations, we investigated the shift between the vertical transition energy ${E}_{calc}^v$ and the band maximum ${E}_{calc}^{\mathrm{max}}$ in the 28 molecules of the Mülheim dataset. Figure 4 illustrates these simulations for three molecules—cyclopropene, pyridine, and pyrimidine (molecules 5, 13, and 15 in Fig. 2).

For energies in eV and absorption cross section in Å^2^.molecule^−1^, the spectrum convolution can be written as (see derivation in Appendix A)

13 $$ {\sigma}_A(E)=0.619n\sum \limits_i\frac{f_i}{\Gamma_i}{e}^{-{\left(E-{E}_i^v+{\delta}_i\right)}^2/{\Gamma}_i^2}, $$

where *n* is the refractive index, and ${E}_i^v$, *f*_*i*_, *δ*_*i*_, and Γ_*i*_ are, respectively, the vertical excitation, oscillator strength, shift, and width of band *i*. We used this expression to fit the simulated spectra. In the fitting process, ${E}_i^v$ and *f*_*i*_ were kept fixed, and we got *δ*_*i*_, and Γ_*i*_. In some cases, the oscillator strength was also optimized to improve the fitting (see supporting information). The number of Gaussian functions used to fit each spectrum was determined by the number of vertical excitations with considerable oscillator strength in the spectral region of interest. Thus, for some bands, several Gaussian functions (up to five) were needed. This is the case of cyclopropene shown in Fig. 4-top (which required three Gaussian functions) and of pyridine in Fig. 4-middle (two Gaussian functions). Other bands, like those in pyrimidine (Fig. 4-bottom), required only one Gaussian function.

The shift results for all molecules are collected in Table 2. All fitted parameters are given in the supporting information. As shown in this table, for all 28 molecules we observe sub-bands with a redshift between the band maximum and the vertical excitation. The shift varies from zero to 0.4 eV; its mean value is 0.11 eV, and the standard deviation is 0.08 eV. As expected, the shift value does not show any correlation either with ${E}_i^v$ or with *f*_*i*_.

**Table 2:** Vertical excitation energy (${E}_i^v$ in eV) and oscillator strengths (*f*_*i*_) for all molecules 28 molecules in the Mülheim dataset in the fitted spectral region. The peak of each Gaussian sub-band is redshifted by *δ*_*i*_ (eV) from ${E}_i^v$. The width of the sub-band is Γ_*i*_ (eV). Molecules are numbered as in Fig. 2

Molecule	Method	${E}_i^v$ (eV)	*f*_*i*_	*δ*_*i*_ (eV)	Γ_*i*_ (eV)
1	CC2	7.16 7.91	0.082 0.381	0.07 0.14	0.192 0.605
2	CC2	6.14	0.745	0.13	0.403
3	CC2	5.18	1.265	0.10	0.400
4	CC2	4.53	1.778	0.06	0.329
5	CC2	6.75 7.09 7.40	0.075 0.031 0.058	0.02 0.19 0.14	0.771 0.294 0.287
6	CC2	5.49	0.105	0.09	0.460
7	CC2	5.64 6.09	0.018 0.018	0.07 0.18	0.296 0.356
8	ADC(2)	6.89 7.19	0.078 0.770	0.06 0.15	0.059 0.155
9	CC2	4.79	0.080	0.13	0.360
10	CC2	6.39 6.43	0.185 0.042	0.10 0.00	0.328 0.749
11	CC2	5.77 5.86 6.28	0.013 0.027 0.189	0.15 0.21 0.21	0.400 0.134 0.309
12	CC2	6.30 6.35 6.44	0.032 0.027 0.153	0.03 0.00 0.14	0.032 0.027 0.153
13	CC2	5.04 5.32	0.005 0.032	0.27 0.05	0.216 0.278
14	CC2	4.18 5.14	0.006 0.085	0.14 0.05	0.213 0.229
15	CC2	4.44 5.52 6.11	0.006 0.030 0.004	0.07 0.11 0.15	0.395 0.262 0.323
16	CC2	3.83 5.39	0.005 0.018	0.08 0.06	0.286 0.359
17	CC2	4.80	0.016	0.06	0.422
18	CC2	5.24	0.056	0.09	0.364
19	CC2	6.38	0.022	0.05	0.128
20	CC2	5.74	0.031	0.08	0.226
21	ADC(2)	5.33	0.593	0.14	0.393
22	CC2	6.13 6.55 6.71 7.23 7.53	0.028 0.021 0.064 0.029 0.337	0.05 0.17 0.01 0.10 0.05	0.349 0.215 0.251 0.241 0.500
23	CC2	5.77 6.36 6.88 7.26 7.60	0.032 0.019 0.019 0.206 0.056	0.00 0.03 0.19 0.09 0.00	0.452 0.239 0.241 0.421 0.240
24	CC2	5.77 6.36 6.76 7.19 7.48	0.026 0.020 0.014 0.189 0.027	0.02 0.08 0.00 0.16 0.00	0.399 0.247 0.259 0.394 0.192
25	CC2	4.68 5.57	0.050 0.137	0.08 0.18	0.412 0.391
26	ADC(2)	5.10 6.10 6.19	0.205 0.028 0.048	0.18 0.41 0.20	0.418 0.275 0.254
27	CC2	5.34 6.30	0.182 0.068	0.17 0.23	0.344 0.430
28	ADC(2)	5.13 5.19	0.247 0.059	0.17 0.00	0.357 0.569

The current results confirm that the redshifts are a common feature shared by many molecules. 80% of the sub-bands presented shifts equal or superior to 0.05 eV. Therefore, it seems that the standard practice of comparing computed vertical excitations to the experimental band maxima should be reconsidered. Ideally, we should compare the experimental band maximum to the calculated band maximum. If only vertical excitations are known (as it is usually the case), a simple estimate of the band maximum is given by ${E}_{calc}^{\mathrm{max}}={E}_i^v-\overline{\delta}$, where $\overline{\delta}=0.1$ eV is the mean value of our benchmark. This point is further elaborated in the next section, where we discuss a specific example.

The fitting of the simulated spectra yielded the sub-band width Γ_*i*_ as a side product. Their values are also given in Table 2. The minimum width is 0.03 eV and the maximum 0.77 eV. The mean value is 0.32 eV, and the standard deviation is 0.14 eV.

### Accounting for the shifts in spectrum convolutions

The parameters in the simple spectrum convolution defined in Eq. (13) are illustrated in Fig. 1. This equation is often used to make simple spectrum simulations from vertical excitation energies and oscillator strengths calculated at the equilibrium geometry. In such applications, the width is either taken as an arbitrary value common to all bands (based on our benchmark, we recommend to employ *Γ* = 0.3 eV) or fitted to experimental values.

Note that the area under each sub-band in Eq. (13) is proportional to the oscillator strength of the corresponding vertical excitation. Nevertheless, it is still common in the literature to find spectrum convolutions taking the oscillator strength as the height of the sub-band. Such a procedure, however, should be avoided, as it does not bear the right functional form *f*_*i*_/Γ_*i*_ and delivers spectra in arbitrary units.

**Fig. 5:**
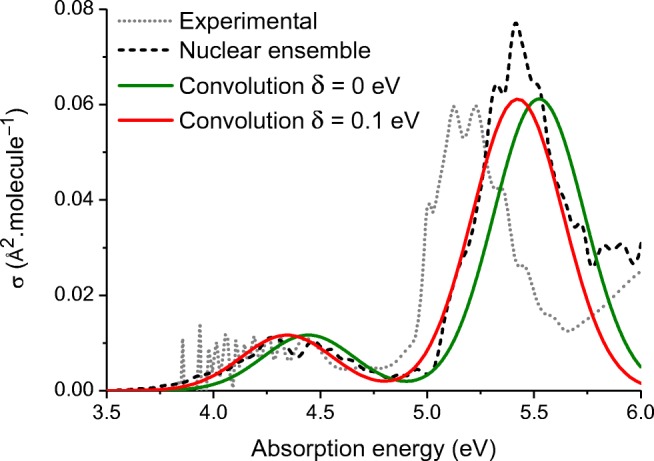
Absorption cross section for pyrimidine in the gas phase. Spectrum convolutions based on Eq. (13) with the same width value for all sub-bands (0.3 eV). Nuclear ensemble spectra computed with 500 points. Experimental data from Ref. [25]

Figure 5 shows the absorption cross-section of pyrimidine in the gas phase. Compared to the experimental spectrum from Ref. [25], the simulations deliver satisfactory results. The intensity and position of the two bands are semi-quantitatively described. Neither the spectrum convolution nor the nuclear ensemble can describe the vibrational resolution of the bands. Nevertheless, in the nuclear ensemble, the band envelope is a result of the simulation, while in the simple convolution, it is assumed to be a sum of Gaussian functions with an arbitrary width *Γ*. Naturally, there is a large difference in the computational effort invested in the two methods. While the Gaussian convolution only demands the calculation of vertical excitation energies and oscillator strengths for the ground-state equilibrium geometry, the nuclear ensemble needs hundreds of such calculations; in the present case, 500 single points.

Now we turn our attention to the spectrum convolutions and the nuclear ensemble results in Fig. 5, and we can clearly see the effect of the shift. With *δ* = 0, the convolution is blueshifted compared to the nuclear ensemble band. A significantly better agreement is obtained when a shift *δ* = 0.1 eV is uniformly applied to both bands.

Experimentally, the high energy band is peaked at 5.2 eV [25]. The vertical excitation energy computed with CC2 is at 5.52 eV, 0.32 eV above the experimental value. However, as we have been discussing, we should preferentially compare the estimated band maximum to the experimental result. The band maximum computed with the nuclear ensemble, still with CC2, is at 5.43 eV, therefore, 0.23 eV above the experimental value. A simpler estimate of the band maximum is obtained by computing ${E}_i^v-\overline{\delta}$, where $\overline{\delta}=0.1$ eV, the mean shift value obtained from our benchmark. In this case, the band maximum estimate is 5.42 eV, 0.22 eV above the experimental value.

## Conclusions

It is well established in the literature that band maxima of absorption spectra are redshifted in comparison to the corresponding vertical excitations by values reaching up to *δ* = 0.2 eV (assuming that both are computed with the same electronic structure method). Using an analytical model and numerical estimates from low dimensionality model systems, we determined that these shifts are primarily caused by the frequency change between the ground and the excited states in multidimensional systems. The geometry displacement between the ground- and excited-state minima does not cause the shift but may affect its value. Nuclear ensemble simulations for a dataset of 28 small organic molecules completed this analysis by delivering a benchmark of shift values. The mean value of the shift calculated over 60 transitions is 0.11 eV with 0.08 eV standard deviation. The mean value of the band width is 0.32 eV with 0.14 eV standard deviation.

Despite the availability of highly accurate methods for spectrum simulations, it is likely that the workhorse method for routine estimation of the one-photon absorption of molecules will remain the simple spectrum convolution of vertical excitations. Based on the results presented in this paper, we recommend that in such convolutions the *δ* shift should be systematically taken into consideration in the comparison between the experimental band maximum and computed vertical excitations. This can be done, for instance, following the suggestion of Ref. [3] of comparing the vertical excitation to the *M*^1^ moment of the experimental band. Alternatively, it can be simply done by estimating the band maximum by redshifting the vertical excitation by 0.1 eV, the mean value of our benchmark.

### Electronic supplementary material

ESM 1 (PDF 1080 kb)

## Appendix A. Derivation of Eq. (13)

Experimentally, the oscillator strength of an absorption band *i* is given as [26].

14 $$ {f}_i=\frac{10^3\ln (10){m}_ec}{\pi {N}_A{e}^2n}\int {\varepsilon}_i\left(\nu \right) d\nu, $$

where *m*_*e*_ is the electron mass, *c* is the speed of light, *N*_*A*_ is the Avogadro’s number, *e* is the electron charge, and *n* is the refractive index (CGS units). *ε*_*i*_ is the molar absorbance (or extinction coefficient). The integral is done over the absorption frequency *ν*. Supposing that the band has a Gaussian shape with maximum ${\varepsilon}_i^{\mathrm{max}}$ at ${\nu}_i^{\mathrm{max}}$, and width *γ*_*i*_, the integral is

15 $$ \int {\varepsilon}_i\left(\nu \right) d\nu =\int {\varepsilon}_i^{\mathrm{max}}{e}^{-{\left(\nu -{\nu}_i^{\mathrm{max}}\right)}^2/{\gamma}_i^2} d\nu =\sqrt{\pi }{\varepsilon}_i^{\mathrm{max}}{\gamma}_i. $$

Thus, the oscillator strength is

16 $$ {f}_i=\frac{10^3\ln (10){m}_ec}{\sqrt{\pi }{N}_A{e}^2n}{\varepsilon}_i^{\mathrm{max}}{\gamma}_i. $$

Following these definitions, the total simulated spectrum can be written as the sum of all bands

17 $$ {\displaystyle \begin{array}{l}\varepsilon \left(\nu \right)=\sum \limits_i{\varepsilon}_i\left(\nu \right)\\ {}\operatorname{}=\sum \limits_i{\varepsilon}_i^{\mathrm{max}}{e}^{-{\left(\nu -{\nu}_i^{\mathrm{max}}\right)}^2/{\gamma}_i^2}\\ {}\operatorname{}=\frac{\sqrt{\pi }{N}_A{e}^2n}{10^3\ln (10){m}_ec}\sum \limits_i\frac{f_i}{\gamma_i}{e}^{-{\left(\nu -{\nu}_i^{\mathrm{max}}\right)}^2/{\gamma}_i^2}.\end{array}} $$

The absorption cross section is [26]

18 $$ \sigma \left(\nu \right)=\ln (10)\frac{10^3}{N_A}\varepsilon \left(\nu \right)=\frac{\sqrt{\pi }{e}^2n}{m_ec}\sum \limits_i\frac{f_i}{\gamma_i}{e}^{-{\left(\nu -{\nu}_i^{\mathrm{max}}\right)}^2/{\gamma}_i^2}. $$

It is convenient to express the absorption cross section in terms of the absorption energy

19 $$ \sigma (E)=\frac{2{\pi}^{3/2}\mathrm{\hslash}{e}^2n}{m_ec}\sum \limits_i\frac{f_i}{\Gamma_i}{e}^{-{\left(E-{E}_i^{\mathrm{max}}\right)}^2/{\Gamma}_i^2}, $$

where Γ_*i*_ is the energy width of the band and ${E}_i^{\mathrm{max}}$ its energy maximum. As usual, ℏ is the reduced Planck constant.

To account for the shift between the band maximum ${E}_i^{\mathrm{max}}$ and the vertical excitation ${E}_i^v$, it is convenient to introduce an energy-shift parameter *δ*_*i*_ to account for this effect:

20 $$ {E}_i^{\mathrm{max}}={E}_i^v-{\delta}_i. $$

Replacing this equation in Eq. (17) leads to (CGS units)

21 $$ \sigma (E)=\frac{2{\pi}^{3/2}\mathrm{\hslash}{e}^2n}{m_ec}\sum \limits_i\frac{f_i}{\Gamma_i}{e}^{-{\left(E-{E}_i^v+{\delta}_i\right)}^2/{\Gamma}_i^2}. $$

For energies in eV and absorption cross section in Å^2^ molecule^−1^, it results in Eq. (13).

From this result, the molar absorbance is

22 $$ \varepsilon \left[{\mathrm{M}}^{\hbox{-} 1}{\mathrm{cm}}^{\hbox{-} 1}\right]=\frac{\sigma \left[{\overset{{}^{\circ}}{\mathrm{A}}}^2.{\mathrm{molecule}}^{\hbox{-} 1}\right]}{3.82353\times {10}^{-5}}. $$
