# Supplementary material for: On the origin of the shift between vertical excitation and band maximum in molecular photoabsorption
Source: J Mol Model. 2020 Apr 21;26(5):107. doi: 10.1007/s00894-020-04355-y (PMC7174274; doi:10.1007/s00894-020-04355-y)
Supplement: Supplementary file 1 — (PDF 1080 kb) [file 894_2020_4355_MOESM1_ESM.pdf]

Supporting Information

On the Origin of the Shift Between Vertical Excitation and Band Maximum in Molecular Photoabsorption

Shuming Bai, Ritam Mansour, Ljiljana Stojanović, Josene M. Toldo, Mario Barbatti

Table of Contents

NUCLEAR ENSAMBLE SPECTRUM SIMULATION AND VERTICAL EXCITION 2
CARTESIAN COORDINATES 10

Nuclear ensemble spectrum simulation and vertical excitations

The nuclear ensemble spectrum for each of the molecules in the Mülheim molecular dataset was computed for N_f states. The spectrum was then fitted with the equation

$$\sigma_A(E) = 0.619\sum_{i=1}^{N}\frac{f_i}{\Gamma_i}e^{-\left(E-E_i^v+\delta_i\right)^2/\Gamma_i^2},$$

where E_i^v, f_i, δ_i, and Γ_i are, respectively, the vertical excitation, oscillator strength, shift, and width of sub-band *i*. In this equation, energies *E* in eV yield absorption cross sections in Å^2.molecule^{-1}. The number *N* of sub-bands was determined by the number of vertical excitations with the largest oscillator strengths lying within the spectral region to be fitted. In the fitting process, E_i^v (eV) and f_i were kept fixed. Γ_i (eV) and δ_i (eV) are the results of the fitting. For some of the molecules, f_i was further released to improve the fitting, producing f_i^{fit}. One band with spectral band maximum $E^{\max}$ (eV) may be composed of one or more sub-bands.

Mol. (N_f)	Spectrum convolution	E_i^v	f_i	δ_i	Γ_i	f_i^{fit}	$E^{\max}$
1 (6)	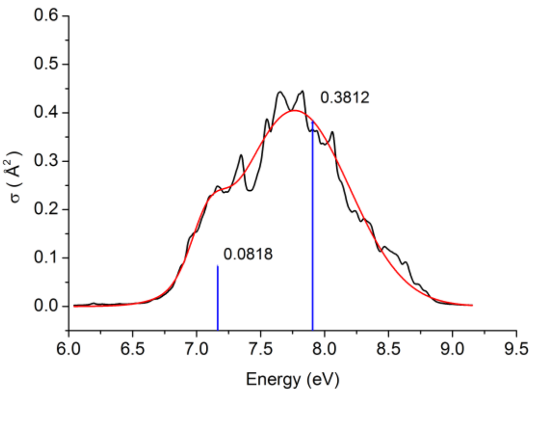	7.16<br>7.91	0.082<br>0.381	0.07<br>0.14	0.192<br>0.605	0.032<br>0.395	 7.77

2<br>(6)	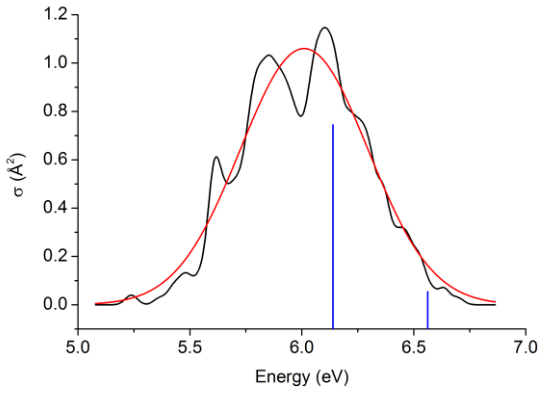	6.14	0.745	0.13	0.403	0.690	6.01
3<br>(6)	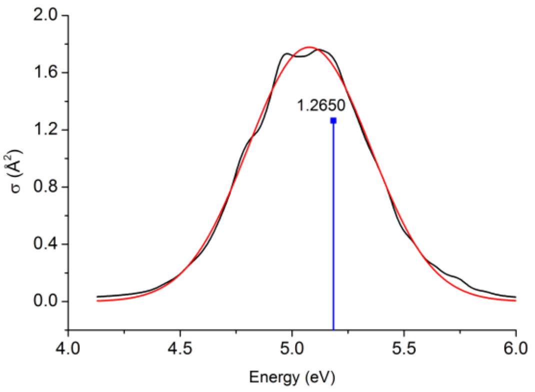	5.18	1.265	0.10	0.400	1.110	5.08
4<br>(6)	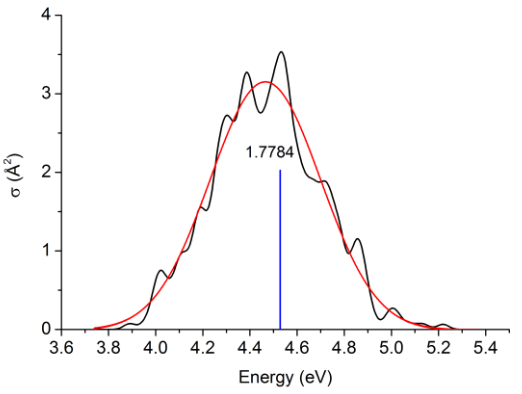	4.53	1.778	0.06	0.329	1.681	4.47
5<br>(6)	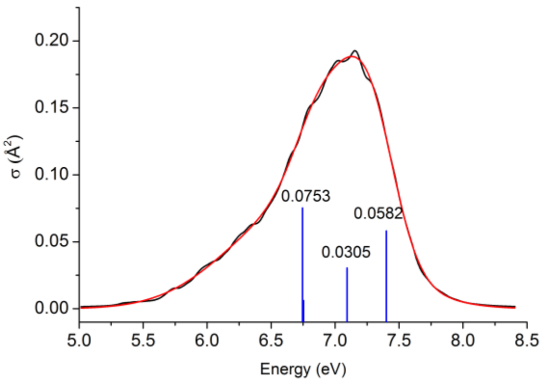	6.75<br>7.09<br>7.40	0.075<br>0.031<br>0.058	0.02<br>0.19<br>0.14	0.771<br>0.294<br>0.287	0.094<br>0.034<br>0.052	7.12

6<br>(6)	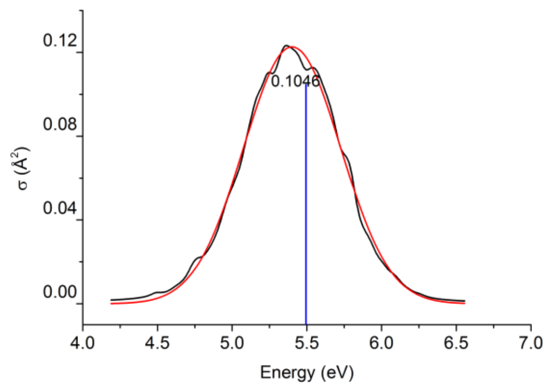	5.49	0.105	0.09	0.460	0.091	5.40
7<br>(6)	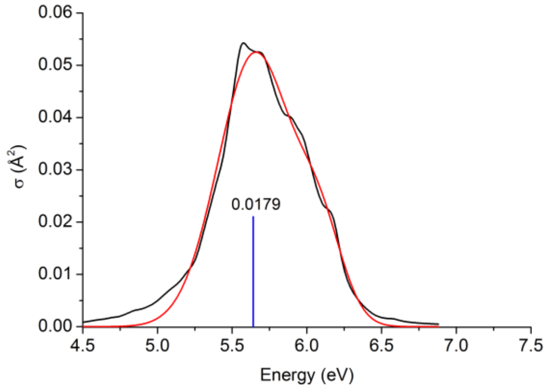	5.64<br>6.09	0.018<br>0.018	0.07<br>0.18	0.296<br>0.356	0.018<br>0.017	5.67
8<br>(10)	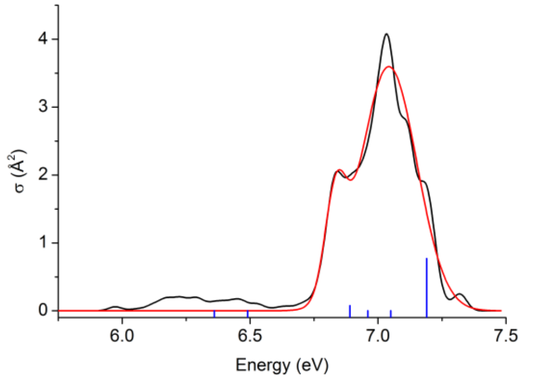	6.89<br>7.19	0.078<br>0.77	0.06<br>0.15	0.059<br>0.155	0.1353<br>0.898	 7.03
9<br>(6)	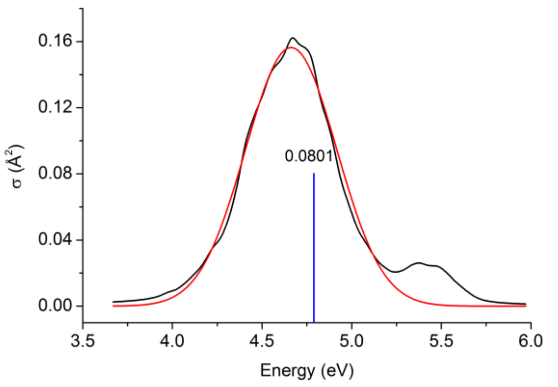	4.79	0.08	0.13	0.360	0.091	4.66

10<br>(6)	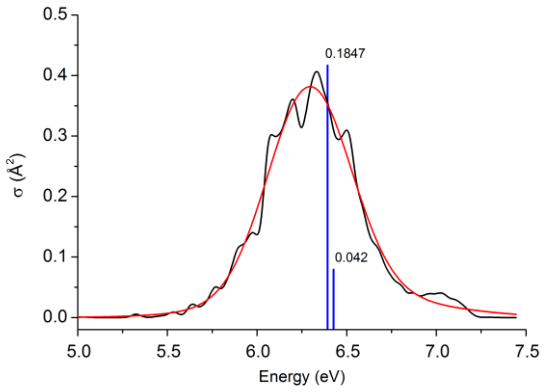	6.39<br>6.43	0.185<br>0.042	0.10<br>0.00	0.328<br>0.749	0.185<br>0.042	6.29
11<br>(6)	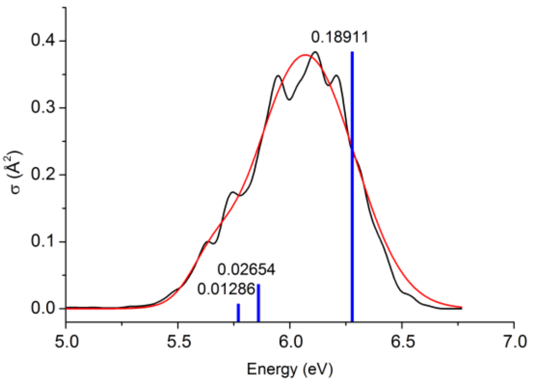	5.77<br>5.86<br>6.28	0.013<br>0.027<br>0.189	0.15<br>0.21<br>0.21	0.4<br>0.134<br>0.309	0.000<br>0.010<br>0.189	6.07
12<br>(6)	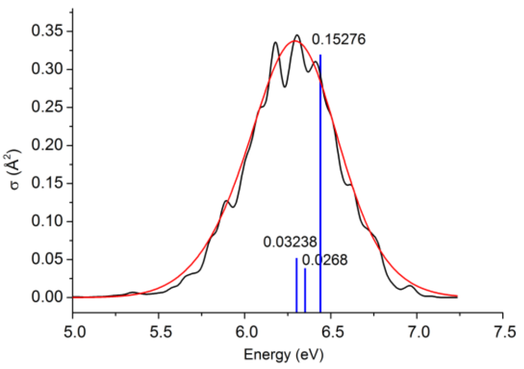	6.30<br>6.35<br>6.44	0.032<br>0.027<br>0.153	0.03<br>0.00<br>0.14	0.032<br>0.027<br>0.153	0.277<br>0.271<br>0.455	6.28
13<br>(6)	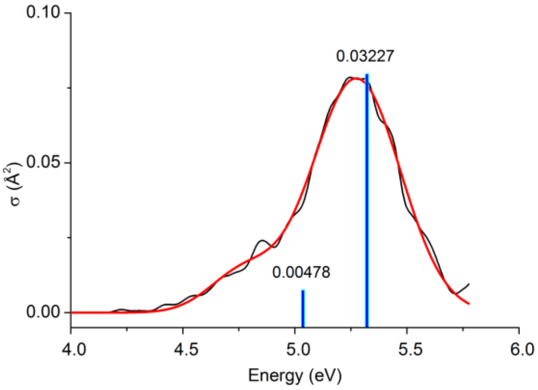	5.04<br>5.32	0.005<br>0.032	0.27<br>0.05	0.216<br>0.278	0.005<br>0.035	5.27

14<br>(6)	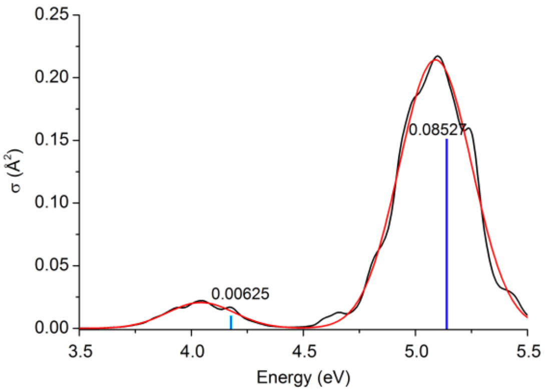	4.18<br>5.14	0.006<br>0.085	0.14<br>0.05	0.213<br>0.229	0.007<br>0.079	4.04<br>5.09
15<br>(6)	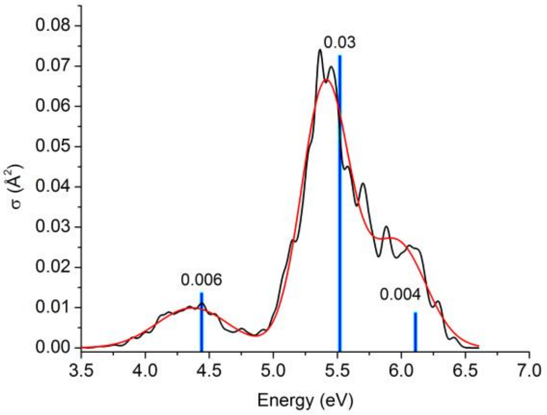	4.44<br>5.52<br>6.11	0.006<br>0.030<br>0.004	0.07<br>0.11<br>0.15	0.395<br>0.262<br>0.323	0.006<br>0.028<br>0.017	4.37<br>5.43
16<br>(6)	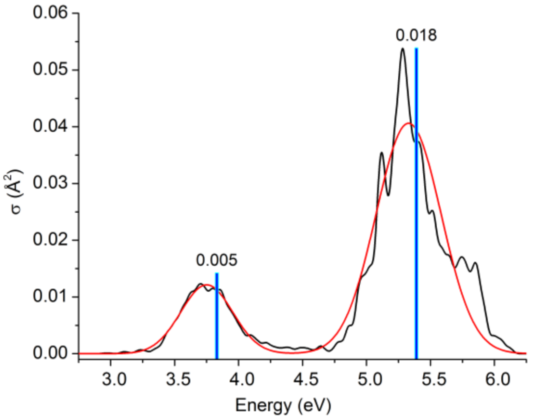	3.83<br>5.39	0.005<br>0.018	0.08<br>0.06	0.286<br>0.359	0.006<br>0.024	3.75<br>5.33

17 (6)	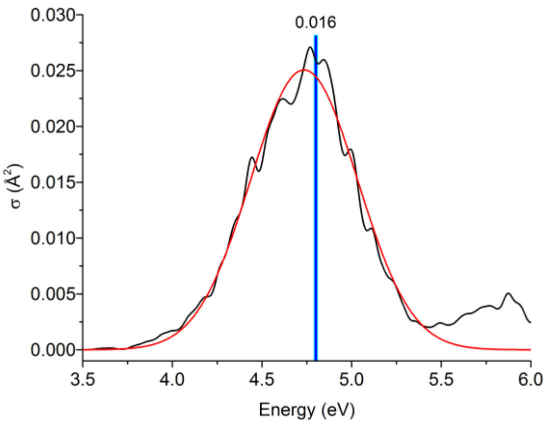	4.80	0.016	0.006	0.422	0.017	4.74
18 (6)	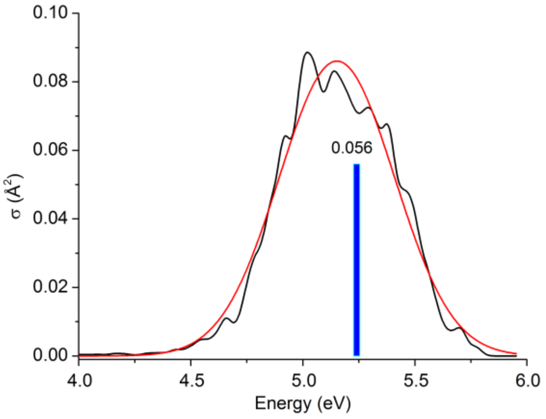	5.24	0.056	0.09	0.364	0.051	5.15
19 (6)	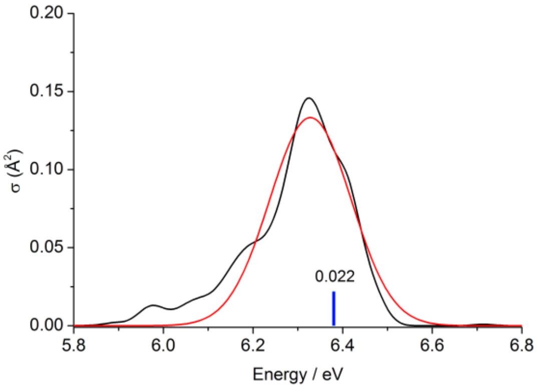	6.38	0.022	0.05	0.128	0.028	6.33
20 (6)	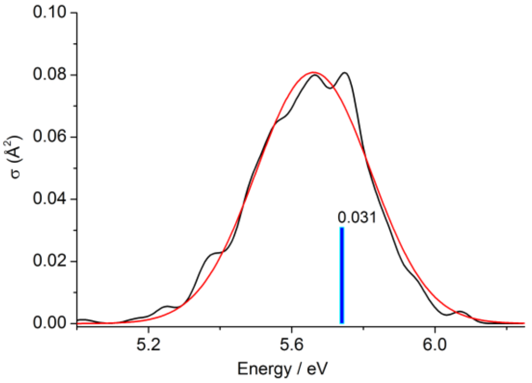	5.74	0.031	0.08	0.226	0.0295	5.66

21	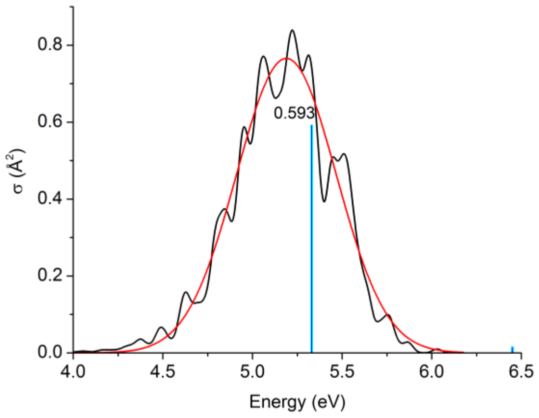	5.33	0.593	0.14	0.393	0.486	5.18
(6)							
22	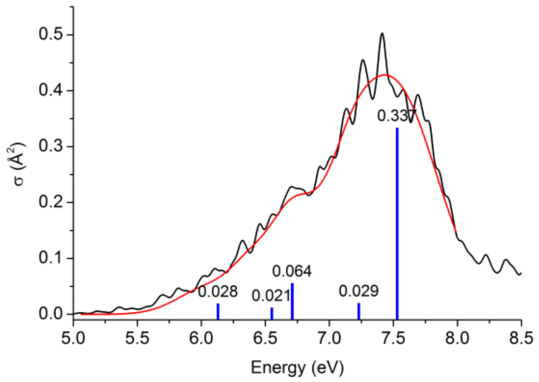	6.13	0.028	0.05	0.349	0.028	
(15)		6.55	0.021	0.17	0.215	0.021	
		6.71	0.064	0.01	0.251	0.064	
		7.23	0.029	0.10	0.241	0.029	
		7.53	0.337	0.05	0.500	0.337	7.43
23	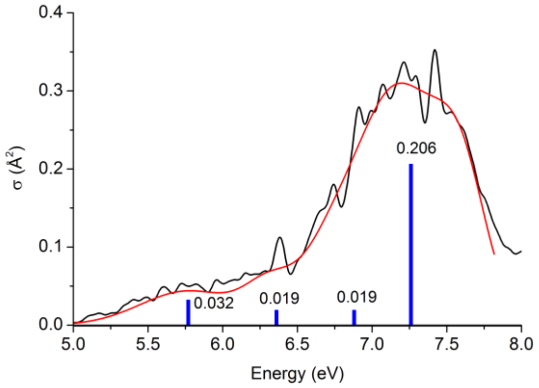	5.77	0.032	0.00	0.452	0.032	
(15)		6.36	0.019	0.03	0.239	0.019	
		6.88	0.019	0.19	0.241	0.019	
		7.26	0.206	0.09	0.421	0.206	7.17
		7.60	0.056	0.00	0.240	0.056	
24	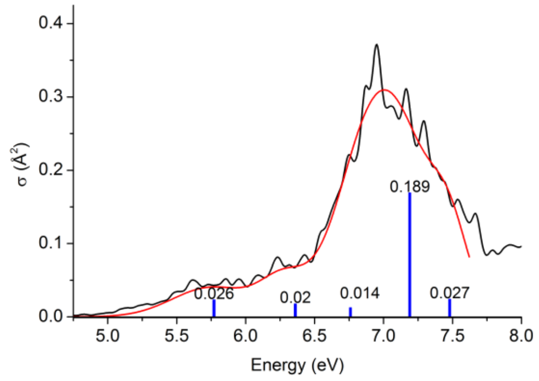	5.77	0.026	0.02	0.399	0.026	
(15)		6.36	0.020	0.08	0.247	0.020	
		6.76	0.014	0.00	0.259	0.014	
		7.19	0.189	0.16	0.394	0.189	6.88
		7.48	0.027	0.00	0.192	0.027	

25<br>(6)	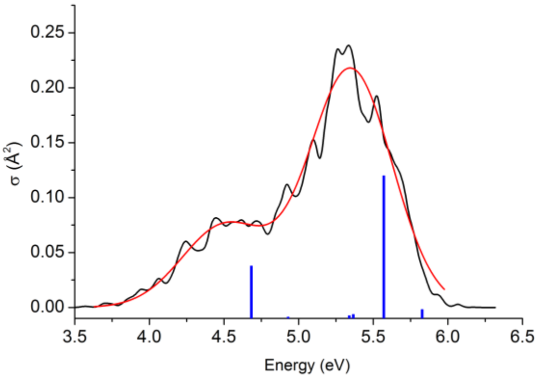	4.68<br>5.57	0.05<br>0.137	0.08<br>0.18	0.412<br>0.391	0.050<br>0.137	 5.35
26<br>(6)	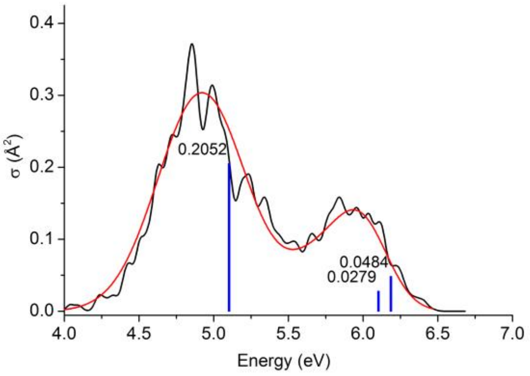	5.10<br>6.10<br>6.19	0.205<br>0.028<br>0.048	0.18<br>0.41<br>0.20	0.418<br>0.275<br>0.254	0.205<br>0.028<br>0.048	4.93
27<br>(6)	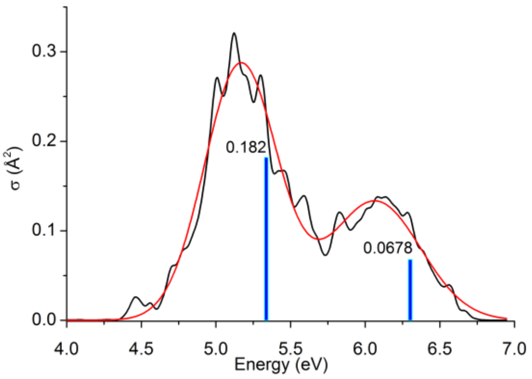	5.34<br>6.30	0.182<br>0.068	0.17<br>0.23	0.344<br>0.430	0.159<br>0.093	5.16
28<br>(6)	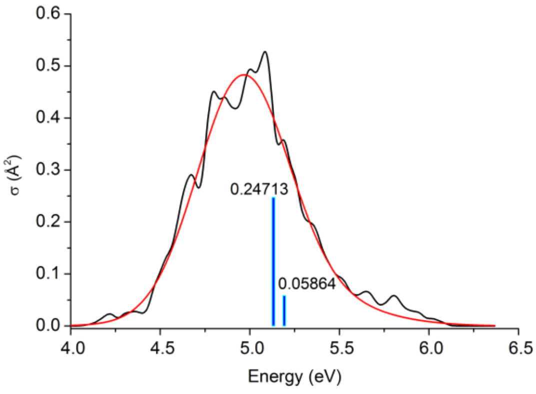	5.13<br>5.19	0.247<br>0.059	0.17<br>0.00	0.357<br>0.569	0.247<br>0.059	4.98

Cartesian coordinates

All coordinates in Angstrom.

```
6
Molecule 1
H     0.0000000    0.9232467    1.2293590
H     0.0000000   -0.9232467    1.2293590
H    -0.0000000    0.9232467   -1.2293590
H    -0.0000000   -0.9232467   -1.2293590
C    -0.0000000    0.0000000    0.6675732
C     0.0000000   -0.0000000   -0.6675732
```

```
10
Molecule 2
H     1.0716094   -2.5511276   -0.0000003
H    -1.0716095    2.5511281   -0.0000001
H     2.0952751   -1.0081485    0.0000003
H    -2.0952755    1.0081484    0.0000001
H    -0.9688536   -1.2159346   -0.0000002
H     0.9688540    1.2159347   -0.0000001
C     0.0006529    0.7263744   -0.0000001
C    -0.0006547   -0.7263736    0.0000013
C     1.1170725   -1.4719244   -0.0000006
C    -1.1170706    1.4719231   -0.0000004
```

```
14
Molecule 3
H    -0.9525277    1.2011149    0.0000004
H     0.9525267   -1.2011153   -0.0000004
H     2.1466767    0.9483596   -0.0000005
H    -2.1466844   -0.9483538    0.0000000
H     2.1222281    3.3884926    0.0000003
H    -2.1222352   -3.3884984   -0.0000006
H     0.2712134    3.3813557    0.0000005
H    -0.2712065   -3.3813613    0.0000003
C    -0.0010365    0.6767206   -0.0000004
C     0.0010410   -0.6767142    0.0000006
C     1.2008285    1.4800638    0.0000011
C    -1.2008236   -1.4801147   -0.0000014
C     1.2002189    2.8258219   -0.0000013
C    -1.2002195   -2.8257713    0.0000013
```

```
18
Molecule 4
H     0.9663455    1.2176794    0.0000016
H    -0.9663493   -1.2176814    0.0000018
H    -2.0920308    0.9800199   -0.0000005
H     2.0920347   -0.9800197   -0.0000015
H    -0.1468336    3.4046013   -0.0000002
H     0.1468219   -3.4046052   -0.0000009
H    -2.1821816    4.7530984    0.0000016
H     2.1821827   -4.7531093    0.0000030
H    -3.2133565    3.2150322   -0.0000006
H     3.2133672   -3.2150287   -0.0000012
C     0.0009424    0.7185001    0.0000046
C    -0.0009259   -0.7185050    0.0000030
C     1.1249822   -1.4757392   -0.0000036
C    -1.1250038    1.4757540   -0.0000015
C     1.1175711   -2.9192987   -0.0000039
C    -1.1175144    2.9192693   -0.0000005
C     2.2328780   -3.6739928    0.0000012
C    -2.2329297    3.6740255   -0.0000023
```

```
7
Molecule 5
H     0.9127303   -0.0000001    1.4510015
H    -0.9127304    0.0000000    1.4510016
H    -0.0000003   -1.5803835   -1.0358614
H     0.0000002    1.5803835   -1.0358616
C     0.0000001   -0.0000000    0.8638999
C     0.0000008   -0.6512093   -0.4980231
C    -0.0000006    0.6512094   -0.4980231
```

```
11
Molecule 6
H    -0.8772743    0.0000001    1.8689369
H     0.8772739   -0.0000003    1.8689366
H     0.0000001    2.2059727    0.6101774
H     0.0000003   -2.2059725    0.6101774
H     0.0000001    1.3477836   -1.8794144
H    -0.0000001   -1.3477836   -1.8794142
C    -0.0000003    0.0000001    1.2141704
C    -0.0000005   -1.1763050    0.2869545
C     0.0000010    1.1763049    0.2869540
C     0.0000011   -0.7309867   -0.9928428
C    -0.0000013    0.7309867   -0.9928422
```

```
15
Molecule 7
H     0.9024903    0.0000001    1.9591074
H    -0.9024910   -0.0000001    1.9591075
H    -0.0000002    2.1509860    0.6161901
H    -0.0000001   -2.1509860    0.6161901
H     1.9193699    1.3357038   -1.0212815
H    -1.9193688   -1.3357025   -1.0212802
H    -1.9193689    1.3357027   -1.0212803
H     1.9193700   -1.3357040   -1.0212816
C     0.0000004    0.0000000    1.3474477
C     0.0000004    1.1201914    0.2735412
C     0.0000006   -1.1201914    0.2735412
```

```
12
Molecule 8
H    -0.0000000    2.4750393   -0.0000003
H     0.0000000   -2.4750393    0.0000004
H     2.1434469    1.2375192    0.0000002
H    -2.1434469   -1.2375192   -0.0000003
H    -2.1434469    1.2375193    0.0000002
H     2.1434469   -1.2375193   -0.0000002
C     0.0000000    1.3936079    0.0000009
C    -0.0000000   -1.3936079   -0.0000011
C     1.2068995    0.6968038   -0.0000009
C    -1.2068995   -0.6968038    0.0000010
C    -1.2068995    0.6968039   -0.0000009
```

```
C     1.2343141    0.6721548   -0.5155031
C    -1.2343154   -0.6721611   -0.5155044
C    -1.2343151    0.6721610   -0.5155042
C     1.2343139   -0.6721547   -0.5155029
```

```
C     1.2068995   -0.6968039    0.0000009
*optimized at RI-MP2/cc-pVTZ level
```

```
18
Molecule 9
H     1.2387361    2.4852891   -0.0000002
H    -1.2387361   -2.4852892    0.0000003
H    -1.2387361    2.4852891   -0.0000003
H     1.2387362   -2.4852893    0.0000003
H     3.3702322    1.2425151    0.0000001
H    -3.3702322   -1.2425151    0.0000002
H    -3.3702322    1.2425151   -0.0000001
H     3.3702322   -1.2425150   -0.0000003
C     0.0000000    0.7154392   -0.0000013
C    -0.0000000   -0.7154393    0.0000016
C     1.2395355    1.4017778    0.0000008
C    -1.2395355   -1.4017777   -0.0000004
C    -1.2395354    1.4017778    0.0000011
C     1.2395354   -1.4017777   -0.0000014
C     2.4302135    0.7063576   -0.0000007
C    -2.4302135   -0.7063576   -0.0000009
C    -2.4302136    0.7063576    0.0000001
C     2.4302135   -0.7063576    0.0000010
```

```
9
Molecule  10
H    -0.0000016    2.0473103    0.8448067
H     0.0000022   -2.0473087    0.8448472
H    -0.0000109    1.3707890   -1.8152153
H     0.0000108   -1.3708283   -1.8152150
C     0.0000081    1.0946836    0.3475987
C    -0.0000085   -1.0946645    0.3475732
C     0.0000040    0.7144683   -0.9628240
C    -0.0000037   -0.7144847   -0.9627598
O    -0.0000004    0.0000072    1.1668186
```

```
10
Molecule 11
H    -0.0000005    2.1083509    0.7688032
H    -0.0000004   -2.1083512    0.7688032
H     0.0000004    1.3576204   -1.8447764
H     0.0000004   -1.3576204   -1.8447765
H     0.0000017    0.0000001    2.1246750
C     0.0000020    1.1246561    0.3336327
C     0.0000019   -1.1246564    0.3336323
C    -0.0000006    0.7100398   -0.9849131
C    -0.0000009   -0.7100398   -0.9849128
N    -0.0000038    0.0000004    1.1198566
```

```
9
Molecule 12
H    -0.0000004    2.1139070    0.7122962
H     0.0000002    1.2013347   -1.8988291
H     0.0000004   -2.0977404    0.6631097
H    -0.0000007   -0.0101215    2.1118901
C     0.0000008    1.1192313    0.3047695
C    -0.0000011    0.6359040   -0.9834410
C    -0.0000018   -1.0904628    0.2837659
N     0.0000008   -0.7421553   -0.9927654
N     0.0000018   -0.0005283    1.1056377
```

```
11
Molecule 13
H    -0.0000001    2.0559118    1.3028845
H    -0.0000000   -2.0559118    1.3028846
H    -0.0000000    2.1518767   -1.1812720
H     0.0000001   -2.1518767   -1.1812720
H    -0.0000001   -0.0000000   -2.4677547
C     0.0000007    1.1428147    0.7195745
C    -0.0000001   -1.1428147    0.7195745
C    -0.0000006    1.1971585   -0.6736021
C    -0.0000003   -1.1971585   -0.6736021
C     0.0000006    0.0000000   -1.3861630
N    -0.0000001    0.0000000    1.4258934
```

```
10
Molecule 14
H    -0.0000006    2.0636968    1.2508116
H     0.0000003   -2.0636965    1.2508115
H     0.0000006    2.0636960   -1.2508112
H    -0.0000003   -2.0636968   -1.2508118
C    -0.0000017    1.1328034    0.6970646
C     0.0000018   -1.1328076    0.6970604
C     0.0000017    1.1328058   -0.6970641
C    -0.0000018   -1.1328050   -0.6970608
N     0.0000002    0.0000035    1.4170793
N    -0.0000002    0.0000004   -1.4170794
```

```
10
Molecule 15
H    -0.0000003    2.1487345    1.1194143
H     0.0000003   -2.1487345    1.1194143
H     0.0000001    0.0000000   -2.3909966
H    -0.0000000    0.0000000    2.4340434
C    -0.0000017    1.1849441    0.6244814
C    -0.0000002   -1.1849442    0.6244814
C     0.0000015    0.0000000   -1.3081913
C     0.0000018    0.0000000    1.3536021
N     0.0000005    1.2047540   -0.7183218
N    -0.0000020   -1.2047541   -0.7183218
```

```
10
Molecule 16
H     0.0000000    2.4009899   -0.1465016
H    -0.0000000   -2.4009899   -0.1465016
H    -0.0000000    1.2683224    2.0975182
H     0.0000000   -1.2683224    2.0975182
C    -0.0000001    1.3220461   -0.0625706
C     0.0000001   -1.3220461   -0.0625706
C    -0.0000012    0.6922491    1.1826742
C     0.0000012   -0.6922491    1.1826742
N     0.0000013    0.6725719   -1.2368636
N    -0.0000013   -0.6725719   -1.2368636
```

9<br>Molecule 17<br>H 0.0000000 0.0000000 2.3777587<br>H 0.0000000 -2.0592001 -1.1888797<br>H 0.0000000 2.0592002 -1.1888797<br>C -0.0000002 0.0000000 1.2949728<br>C -0.0000002 1.1214797 -0.6474859<br>C -0.0000002 -1.1214797 -0.6474860<br>N 0.0000002 -0.0000001 -1.3812197<br>N 0.0000002 1.1961723 0.6906109<br>N 0.0000002 -1.1961723 0.6906108	8<br>Molecule 18<br>H -0.0000001 0.0000000 -2.3443429<br>H -0.0000000 0.0000000 2.3443429<br>C -0.0000012 -0.0000002 1.2633169<br>C -0.0000011 -0.0000002 -1.2633169<br>N 0.0000016 1.2038705 0.6690823<br>N -0.0000003 -1.2038703 0.6690824<br>N -0.0000004 1.2038704 -0.6690823<br>N 0.0000015 -1.2038703 -0.6690824
4<br>Molecule 19<br>H -0.0000000 0.9345111 -0.5851088<br>H -0.0000000 -0.9345111 -0.5851089<br>C 0.0000000 -0.0000000 -0.0011336<br>O -0.0000000 0.0000001 1.2162993	10<br>Molecule 20<br>H 0.2672357 2.1177411 -0.0811858<br>H -0.2706580 -2.1191451 0.0050999<br>H -0.7931082 1.3992524 -1.3165325<br>H 0.8095583 -1.4556420 -1.2434852<br>H -0.9237166 -1.2343667 -1.3998431<br>H 0.9426138 1.1750095 -1.4369899<br>C -0.0010054 0.0018813 0.0955916<br>C 0.1165675 1.2610620 -0.7306552<br>C -0.1086545 -1.2912054 -0.6780215<br>O -0.0085518 0.0277723 1.3207213
12<br>Molecule 21<br>H 0.0000494 2.1750309 1.2559309<br>H -0.0000624 -2.1750410 1.2559394<br>H -0.0000456 2.1749964 -1.2559163<br>H 0.0000663 -2.1750070 -1.2559245<br>C 0.0000074 -0.0000012 1.4399660<br>C -0.0000098 -0.0000015 -1.4400032<br>C 0.0000311 1.2623586 0.6743512<br>C -0.0000265 -1.2623498 0.6743014<br>C -0.0000163 1.2623125 -0.6743359<br>C 0.0000208 -1.2623029 -0.6742857<br>O 0.0000182 0.0000024 2.6774407<br>O -0.0000328 0.0000025 -2.6774640<br>*optimized at RI-MP2/cc-pVTZ level	6<br>Molecule 22<br>H -0.9181389 -0.6047967 0.0000001<br>H 1.0604197 -1.7752852 -0.0000009<br>H 2.0213846 -0.3257870 -0.0000003<br>C 0.0026261 -0.0007044 0.0000001<br>O -0.0004313 1.2227863 -0.0000004<br>N 1.1211160 -0.7739624 0.0000015
9<br>Molecule 23<br>C -1.4535385 -0.1460459 -0.0137572<br>C 0.0435384 0.0674388 -0.0036137<br>N 0.7882631 -1.0802540 -0.0300894<br>O 0.5565716 1.1831450 -0.0251860<br>H 0.3737140 -1.9782431 0.1354097<br>H 1.7877234 -0.9870211 0.0305193<br>H -1.9256843 0.6889138 0.4937899<br>H -1.7498349 -1.0804199 0.4595219<br>H -1.7987483 -0.1584021 -1.0468075	12<br>Molecule 24<br>C -2.5156437 0.0291523 0.0032803<br>C -1.2688900 -0.8368858 -0.0195522<br>C 0.0003103 -0.0033223 -0.0583083<br>N 1.1564389 -0.7346161 -0.1367601<br>O 0.0050363 1.2233025 0.0142435<br>H 1.1301933 -1.7058354 -0.3903800<br>H 2.0111614 -0.2257292 -0.2875381<br>H -1.2218126 -1.4778825 0.8636852<br>H -1.2715911 -1.5046319 -0.8844989<br>H -3.4116558 -0.5879151 0.0446155<br>H -2.5641817 0.6559302 -0.8843390<br>H -2.5040564 0.6898574 0.8666003
13<br>Molecule 25<br>H -2.1065431 -1.4317395 0.0000015<br>H -0.1704627 -2.7983215 0.0000006<br>H 2.0671476 -1.6557616 0.0000002<br>H 3.1646905 0.5575736 -0.0000023<br>H 2.2294828 2.0273267 -0.0000011	15<br>Molecule 26<br>H 0.2122931 -2.6709033 0.0000020<br>H 2.0472045 0.9214924 0.0000007<br>H -1.9374296 -1.7007677 0.0000012<br>H -3.3538478 0.3072525 -0.0000000<br>H -2.6130697 1.6606002 0.8746831

C -0.0614403 -1.7230070 0.0000026<br>C 1.1458836 -1.0955859 -0.0000016<br>C 1.1088609 0.3366400 -0.0000024<br>C -1.2231779 0.4320932 0.0000003<br>O -2.3114325 0.9979696 -0.0000012<br>N -0.0008648 1.0579731 0.0000036<br>N -1.1988996 -0.9901176 -0.0000044<br>N 2.2778551 1.0234108 0.0000042	H -2.6130698 1.6605993 -0.8746830<br>C 1.3549915 -0.9933332 -0.0000021<br>C -0.0035506 1.1206863 -0.0000020<br>C -1.2135013 0.3088024 -0.0000005<br>C -1.0817869 -1.0395569 0.0000025<br>C -2.5279876 1.0167456 0.0000005<br>O 2.4400423 -1.5594333 0.0000027<br>O 0.0215293 2.3508550 0.0000011<br>N 0.1432358 -1.6653702 -0.0000068<br>N 1.1906971 0.3811858 0.0000007<br>*optimized at RI-MP2/cc-pVTZ level
12<br>Molecule 27<br>H -2.0190306 -1.5179910 -0.0000013<br>H -0.0217045 1.9899598 -0.0000013<br>H 2.1741287 -1.6030759 0.0000000<br>H -0.0257062 -2.7824323 -0.0000006<br>C -1.2377676 0.3594731 -0.0000017<br>C 1.2809741 0.3891476 0.0000018<br>C 1.2433659 -1.0615108 0.0000021<br>C 0.0530383 -1.7049613 -0.0000019<br>O -2.3080074 0.9510125 0.0000010<br>O 2.2875846 1.0917236 -0.0000021<br>N -1.1387795 -1.0271625 0.0000021<br>N -0.0006569 0.9775873 0.0000018	15<br>Molecule 28<br>H 0.9724910 -3.0652695 0.0000008<br>H 2.1282579 2.0673034 0.0000015<br>H 3.3043287 0.7723159 0.0000012<br>H -3.0452884 -0.3331035 -0.0000011<br>H -2.7089700 2.1952694 0.0000005<br>C 0.6633946 -2.0276064 0.0000010<br>C 1.3584129 0.1743808 -0.0000031<br>C 0.0029969 0.5485451 0.0000008<br>C -0.9220597 -0.4996009 -0.0000030<br>C -1.9040277 1.4786901 -0.0000019<br>N -0.6610026 -1.8148417 0.0000022<br>N 1.6729121 -1.1327488 -0.0000016<br>N -2.1481429 0.1262956 0.0000020<br>N -0.6168560 1.7850284 0.0000020<br>N 2.3516071 1.0887607 -0.0000013<br>*optimized at RI-MP2/cc-pVTZ level
